# A case of laboratory-acquired Salmonella Typhi infection due to phage typing in Japan: whole-genome sequencing confirms the source of infection

**DOI:** 10.1099/acmi.0.001121.v4

**Published:** 2026-03-13

**Authors:** Masatomo Morita, Kenichi Lee, Akio Sugiyama, Narumi Kojima, Yasuhiro Kawai, Hidemasa Izumiya, Yukihiro Akeda

**Affiliations:** 1 Department of Bacteriology I, National Institute of Infectious Diseases, Japan Institute for Health Security, Toyama 1-23-1, Shinjuku-ku, Tokyo 162-8640, Japan; 2 Department of Gastroenterology, Ogikubo Hospital, Imagawa 3-1-24, Suginami-ku, Tokyo 167-0035, Japan; 3 Department of Clinical Laboratory, Sainokuni Higashiomiya Medical Center, Toro-cho 1522, Kita-ku,Saitama 331-8577, Japan; 4 Research Center for Biosafety, Laboratory Animal and Pathogen Bank, National Institute of Infectious Diseases, Japan Institute for Health Security, Toyama 1-23-1, Shinjuku-ku, Tokyo 162-8640, Japan

**Keywords:** laboratory-acquired infection, phage typing, *Salmonella* Typhi

## Abstract

**Introduction.** Typhoid fever, caused by *Salmonella enterica* serovar Typhi, is a systemic infection. Approximately 40 cases occur annually in Japan, most of which are imported. In August 2023, a researcher engaged in phage typing of *S*. Typhi was diagnosed with typhoid fever.

**Case Presentation.** A 48-year-old man presented with high fever, diarrhoea, malaise and loss of appetite. Initial findings, including liver dysfunction and severe inflammation, led to a suspected diagnosis of autoimmune disease. However, blood cultures identified *S*. Typhi, confirming typhoid fever. Comparative genomic analysis demonstrated clonality between the strain handled in the laboratory and the patient isolates, indicating a laboratory-acquired infection.

**Conclusion.** This case underscores the need for ongoing vigilance regarding the risk of laboratory-acquired infections and highlights the value of whole-genome sequencing for tracking. It would be also emphasized that this is the first reported case in Japan linked to phage typing, a conventional typing method for *S*. Typhi. This raises the urgency of transitioning from phage typing to genotyping and recommends mandatory typhoid vaccination for laboratory personnel working with *S*. Typhi to minimize infection risk.

## Data Summary

Short-read sequence data are available in the DDBJ Sequenced Read Archive under the accession numbers DRR657151–DRR657161.

## Introduction

Typhoid fever is a systemic infection caused by *Salmonella enterica* subsp. *enterica* serovar Typhi, which has humans as its only host. Improving hygiene infrastructure and ensuring access to safe drinking water significantly reduces the incidence of typhoid fever [1]. Approximately 40 cases are reported annually in Japan, and most cases are associated with recent travel to typhoid-endemic countries (https://www.niid.go.jp/niid/en/idwr-e.html). Isolated *S*. Typhi strains from patients and asymptomatic carriers are submitted to the Department of Bacteriology I at the National Institute of Infectious Diseases (NIID) through prefectural public health institutes or local public health centres. These strains undergo phage typing for epidemiological surveillance. In August 2023, a researcher responsible for phage typing was diagnosed with typhoid fever. Because he had no history of travel to an endemic area, a laboratory-acquired infection (LAI) was suspected. Comparative genomic analysis of the *S*. Typhi strains handled in the laboratory prior to the onset of illness and the isolates from the patient confirmed their clonality. We herein report this case.

## Case presentation

**Fig. 1.**
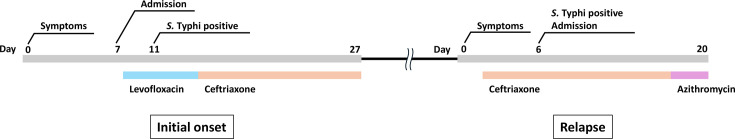
Timeline of symptom onset, microbiological confirmation and antimicrobial therapy during the initial and relapsed episodes of typhoid fever.

In August 2023, a 48-year-old previously healthy man presented to the hospital after 1 week of high-grade fever, diarrhoea, severe malaise and loss of appetite. Laboratory blood tests revealed elevated levels of aspartate transaminase (147 U l^−1^; reference value: 13–30 U l^−1^), alanine transaminase (80 U l^−1^; reference value: 10–42 U l^−1^), lactate dehydrogenase (739 IU l^−1^; reference value: 124–222 IU l^−1^), alkaline phosphatase (217 IU l^−1^; reference value: 38–113 IU l^−1^), gamma-glutamyl transferase (113 U l^−1^; reference value: 13–64 U l^−1^), C-reactive protein (18.06 mg dl^−1^; reference value: 0–0.14 mg dl^−1^) and serum ferritin (5,946 ng ml^−1^; reference value: 21–282 ng ml^−1^). The patient was admitted with a suspected autoimmune disease based on findings of liver dysfunction, severe inflammation, markedly elevated serum ferritin levels and stomatitis. Subsequently, a blood culture on admission became positive for Gram-negative rods, which were identified as *S*. Typhi, leading to a diagnosis of typhoid fever. He was empirically treated with levofloxacin for the first 6 days, followed by ceftriaxone for 14 days. By the 10th day of ceftriaxone treatment, blood cultures turned negative, and the patient was discharged 16 days after admission. Unfortunately, he relapsed with a fever ~2 weeks after completing the ceftriaxone therapy. The isolates from the initial and relapsed episodes exhibited intermediate susceptibility to ciprofloxacin, which was associated with a serine-to-phenylalanine substitution at position 83 in the DNA gyrase A subunit gene (*gyrA*), as identified by ResFinder 4.7.2 using whole-genome sequencing (WGS) data [2,3]. No other known mutations or acquired resistance genes besides the *gyrA* mutation were identified, and they remained susceptible to ceftriaxone. The antimicrobial susceptibility testing was performed at the NIID after completion of the treatment, and the results are summarized in Table 1 [4,5]. These findings suggest that the relapse was not attributable to the mutation in the *gyrA* or to another unidentified mutation associated with drug resistance. Instead, it was more likely due to the limited intracellular penetration of ceftriaxone, which may have allowed *S*. Typhi to persist within host cells. He was re-hospitalized after a repeat blood culture again confirmed the presence of *S*. Typhi. Following another 14-day course of ceftriaxone, blood cultures became negative, and the patient was discharged. To help prevent a further relapse, azithromycin was administered for 3 days post-discharge. The patient has shown no new symptoms, and no secondary infections were reported (Fig. 1).

**Table 1.** Antimicrobial susceptibility of *S*. Typhi strain isolated from the patient

Antimicrobial	MIC (µg/ml)	Interpretation*
Ampicillin	1	Susceptible
Chloramphenicol	2	Susceptible
Tetracycline	0.5	Susceptible
Azithromycin	8	Susceptible
Ceftriaxone	0.06	Susceptible
Imipenem	0.06	Susceptible
Ciprofloxacin	0.5	Intermediate
Trimethoprim/sulfamethoxazole	1.25	Susceptible

*Interpretation of azithromycin susceptibility was based on EUCAST criteria; all other antimicrobials were interpreted according to CLSI guidelines.

**Fig. 2.**
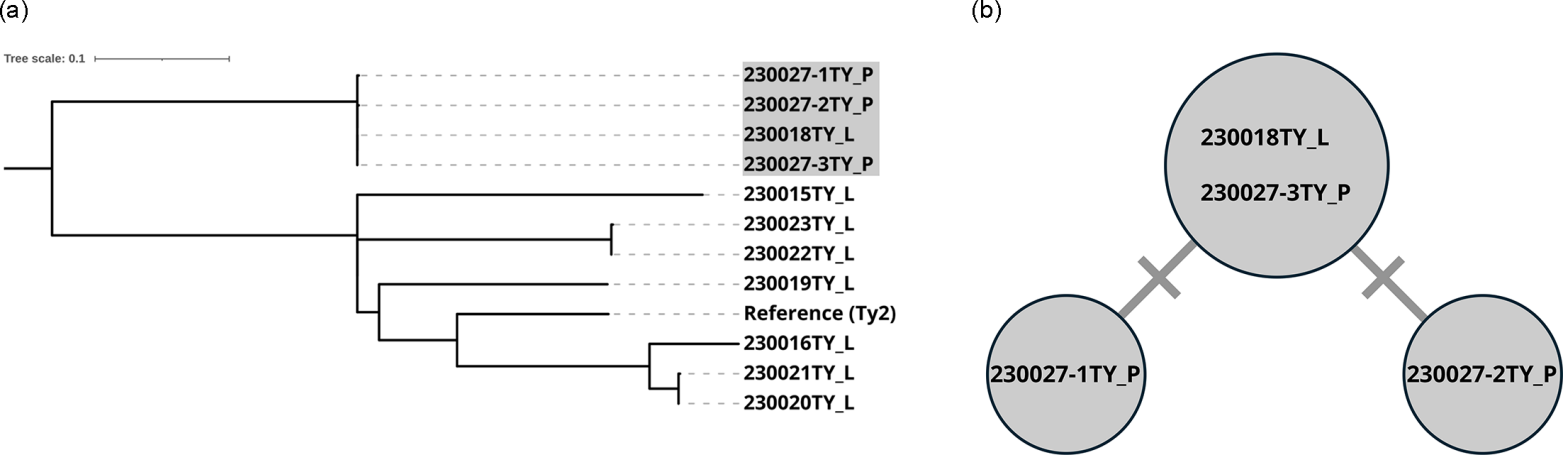
Molecular epidemiological analysis based on core genome SNVs. (**a**) Midpoint-rooted phylogenetic tree of *S*. Typhi strains sequenced in this study, constructed using the genome of *S*. Typhi strain Ty2 as the reference. In total, 879 SNVs were identified on the core genome of 4,231,696 bp. *S*. Typhi strains related to the LAI are highlighted in grey, and source of strain is indicated after the strain name (L; laboratory stock, P; patient). (**b**) Median-joining tree of *S*. Typhi strains associated with the LAI. The number of short lines on the connecting branches between circles indicates the number of SNVs.

The patient had not travelled abroad in the 5 months prior to disease onset, and no epidemiological information was available to suggest a domestic contract. He worked as a researcher in a laboratory at the NIID and was responsible for the phage typing of *S*. Typhi. Although there was no record of a laboratory accident, occupational exposure was investigated as a potential source of infection. The use of WGS in investigating LAIs has been previously reported, and we applied a similar approach in this case [6,8]. The patient had handled eight *S*. Typhi strains during the month preceding symptom onset. To assess the relationship between these laboratory strains and the patient’s isolates, we collected three isolates from the patient and performed phylogenetic analysis based on WGS (Table 2). Genomic DNA libraries were prepared using the QIAseq FX DNA library kit (Qiagen) following the manufacturer’s instructions, and sequencing was carried out using the iSeq 100 or MiSeq platform (Illumina). Core genome single-nucleotide variations were extracted using BactSNP v.1.1.0, with the genome of *S*. Typhi strain Ty2 (GenBank accession number: AE014613) as a reference, and phylogenetic relationships were inferred by constructing a phylogenetic tree using IQ-TREE v.2.1.2 with 1,000 ultrafast bootstrap replicates [9,11]. The phylogenetic tree was visualized using iTOL [12]. The three patient isolates (230027-1TY, 230027-2TY and 230027-3TY) and one laboratory strain (230018TY) formed a distinct cluster, with pairwise SNV differences ranging from 0 to 2, clearly separated from other strains (Fig. 2). We also confirmed that these four isolates possessed *Salmonella* pathogenicity islands SPI-1 to SPI-10 using SPIFinder 2.0 [13]. The patient had handled this strain for phage typing 16 days before the onset of illness, a timeline consistent with the incubation period of typhoid fever. These findings indicated that this strain was the source of infection, confirming an LAI, which was subsequently reported as an incident.

**Table 2.** List of *S*. Typhi strains sequenced in this study

No.	Strain	Phage type	Source	Sequence type*	Remarks
1	230015TY	E12	Laboratory stock	2	Used 16 days before onset
2	230016TY	UVS4	Laboratory stock	1	Used 16 days before onset
3	230018TY	DVS	Laboratory stock	2	Used 16 days before onset, causative strain
4	230019TY	DVS	Laboratory stock	1	Used 16 days before onset
5	230020TY	UVS4	Laboratory stock	1	Used 16 days before onset
6	230021TY	UVS4	Laboratory stock	1	Used 3 days before onset
7	230022TY	UVS4	Laboratory stock	2	Used 3 days before onset
8	230023TY	UVS4	Laboratory stock	2	Used 3 days before onset
9	230027-1TY	DVS	Patient	2	Blood (initial onset)
10	230027-2TY	DVS	Patient	2	Faeces (initial onset)
11	230027-3TY	DVS	Patient	2	Blood (relapse)

*The sequence type was determined using the PubMLST database [24].

## Discussion

Improved biosafety management can reduce but not completely eliminate the risk of LAIs [14,16]. While some cases of *S*. Typhi infection have been attributed to unsafe practices such as mouth pipetting, eating or smoking in the laboratory, other cases have occurred despite adherence to biosafety protocols and in the absence of any identifiable laboratory accidents [7,8, 17,19]. In this case, the laboratory where the patient performed phage typing was a well-maintained biosafety level 2 facility, equipped with certified biosafety cabinets. The patient handled *S*. Typhi inside the biosafety cabinet while wearing laboratory gloves and a lab coat. Although the investigation was unable to determine the exact point of exposure, the phage typing method was identified as having a high risk of infection.

The phage typing method has been used since 1947 as the standard approach for distinguishing strains within the same serovar of *S*. Typhi [20]. In addition to the general risks associated with handling liquid cultures, certain steps in the phage typing procedure may pose an elevated risk of droplet generation. For example, the protocol involves flooding agar plates with bacterial liquid culture to ensure even distribution across the surface of the plate. This step involves tilting and rotating the plate, followed by removing excess culture. These actions may cause droplets that can contaminate gloves, bench surfaces or other equipment and could serve as potential sources of infection. This case highlights the infection risk associated with phage typing, particularly during the handling of liquid cultures.

Continuous vigilance is essential to prevent LAIs, and vaccination against typhoid fever should be mandatory for all personnel working with *S*. Typhi, especially because the patient in this case had not been vaccinated. International guidance from both the World Health Organization and the U.S. Centers for Disease Control and Prevention recommends typhoid vaccination for laboratory personnel handling *S*. Typhi [21,22]. In Japan, no typhoid vaccine was officially approved at the time of this LAI in 2023. Vaccination relied on imported products not covered by vaccine injury compensation programmes. However, in 2024, Japan approved its first typhoid vaccine. It is now both feasible and advisable to align national biosafety policies with international practices by recommending vaccination for personnel handling *S*. Typhi.

This is the first reported typhoid case in Japan associated with phage typing. Although phage typing has a long history, the supply of certified typing phages is now at risk of discontinuation, highlighting the urgent need for alternative approaches. A genotyping method based on WGS of *S*. Typhi has already been established internationally that should be adopted in Japan as well [23]. In Japan, all *S*. Typhi isolates are routinely submitted to the NIID, the national authority responsible for nationwide surveillance. This system provides an ideal setting for implementing WGS-based genotyping, and initial preparations for implementation are in progress.

## Conclusion

This case highlights the risk of LAIs associated with phage typing of *S*. Typhi and underscores the need for continued vigilance. Given the inherent risks of phage typing, there is an urgent need to transition to safer, more advanced methods such as WGS-based genotyping, which offers greater discriminatory power with a lower risk of bacterial exposure. Additionally, mandatory typhoid fever vaccination should be considered for all personnel working with *S*. Typhi to further reduce the risk of LAIs.

## Abbreviations

LAI: laboratory-acquired infection
NIID: National Institute of Infectious Diseases
WGS: whole-genome sequencing
